# 491. Persistence of SARS-CoV-2 Iinfection in Immunocompromised Children

**DOI:** 10.1093/ofid/ofab466.690

**Published:** 2021-12-04

**Authors:** Susan Dolan, Jean Mulcahy Levy, Angela Moss, Kelly Pearce, Molly Butler, Samuel R Dominguez, Samuel R Dominguez, Sarah Jung, Kelly Maloney, Eric Mwangi, Suchitra Rao

**Affiliations:** 1 Children’s Hospital Colorado, Aurora, Colorado; 2 University of Colorado School of Medicine, Aurora, Colorado; 3 University of Colorado, School of Medicine, Aurora, CO; 4 University of Colorado School of Medicine, Denver, CO

## Abstract

**Background:**

The temporal dynamics of SARS-CoV-2 infectivity in immunocompromised children (IC) are unknown but may have important infection control implications. We evaluated SARS-CoV-2 viral persistence and assessed factors associated with viral persistence and cycle threshold (CT) values as a surrogate of viral load for IC.

**Methods:**

We conducted a retrospective cohort study of SARS-CoV-2-positive IC at a large quaternary pediatric hospital from March 2020-2021. Immunocompromised status was defined as primary or secondary/acquired immunodeficiencies due to comorbidities or immunosuppressive treatment. The primary outcome was time to first-of-two consecutively negative SARS-CoV-2 PCR tests ≥ 24 hours apart. Polymerase chain reaction (PCR) testing of sequential patient samples was conducted using the Centers for Disease Control 2019-nCoV Real-Time RT-PCR Diagnostic Panel (CDC assay). Chi-square, Fisher exact, and Wilcoxon tests were used to compare demographic and clinical characteristics. Kaplan-Meier curve median event times and log-rank tests were used to compare outcomes. Subjects without 2 consecutive negative tests censored at the last test. Analyses were conducted using SAS v 9.4.

**Results:**

Ninety-one children met inclusion criteria, and 67 children had more than 1 test (Figure 1). Median age was 15.5 years (IQR 8-18 yrs), 64% were male, 58% of children were white, and 43% were Latinx. Most (67%) were tested in outpatient settings, and 58% of children were asymptomatic. The median time to two negative tests was 42 days (IQR 25.0,55.0), with no difference in duration of positivity with specific diagnoses, degree of lymphopenia, or symptomatic vs asymptomatic illness. Five of 7 (71%) children with samples available for repeat testing had initial C_T_ values < 30, indicating a moderate to high viral load, and of these, 4 (57%) had repeat testing 21 to 30 days later with C_T_ values < 30 (Figure 2), suggesting persistence of moderate to high viral loads.

Figure 1. Plot of immunocompromised children in cohort with positive SARS CoV2 PCR and subsequent testing (n = 67).

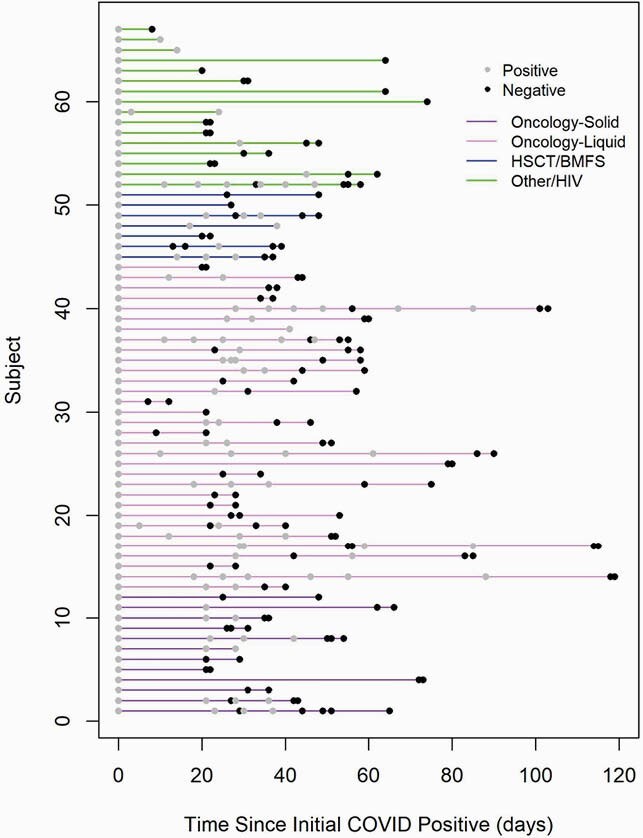

Timelines of immunocompromised children in cohort with positive SARS CoV2 PCR and subsequent testing, grouped by immunocompromising condition. Each line represents an individual patient. Positive results are shown in light grey, negative results are shown in black.

Figure 2. Plot of CT values from SARS-CoV-2 PCR testing over time among children with sequential samples available for retesting (n = 7)

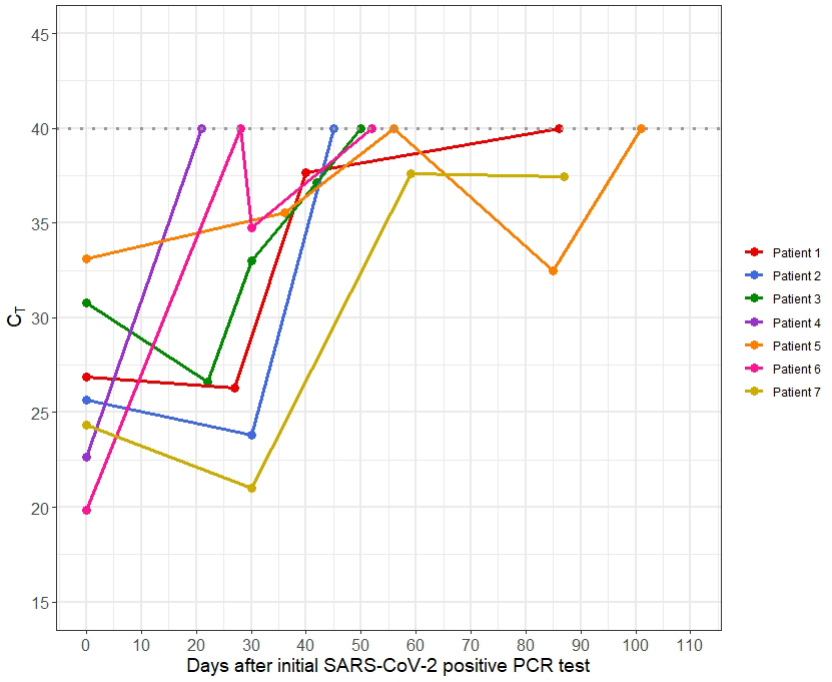

Plot of CT values (y axis) from SARS-CoV-2 PCR testing on the CDC assay over time (x axis) in days from initial positive test. Repeated testing which yielded a negative result on the CDC assay or intermittent negative results on clinical testing represented as CT value of 40. Each line represents a unique patient.

**Conclusion:**

The median duration of viral persistence among IC with SARS-CoV-2 infection was 6 weeks, with no significant difference in immunocompromised diagnoses or clinical presentation, with over half of children with testing on the same platform having moderate to high viral loads after 3 weeks, suggesting potential transmission risk.

**Disclosures:**

**Samuel R. Dominguez, MD, PhD**, **BioFire Diagnostics** (Consultant, Research Grant or Support)**DiaSorin Molecular** (Consultant)**Pfizer** (Grant/Research Support) **Samuel R. Dominguez, MD, PhD**, BioFire (Individual(s) Involved: Self): Consultant, Research Grant or Support; DiaSorin Molecular (Individual(s) Involved: Self): Consultant; Pfizer (Individual(s) Involved: Self): Grant/Research Support **Suchitra Rao, MBBS, MSCS**, **BioFire** (Research Grant or Support)

